# The Basis and Application of Wright’s Opsonin Theory

**Published:** 1907-03

**Authors:** E. Weinstein


### THE BASIS AND APPLICATION OF WRIGHT'S OPSONIN THEORY.

Dr. DR. E. WEINSTEIN (Berliner klinische Wochenschrift, 1906, No. 30, p. 1007).

In 1903 Wright showed that among other bacteriotropic substances one is of special importance. This substance chemically unites with those bacteria which produce infection or immunization, whereby the bacteria are so altered that they become an easy prey for the devouring leucocytes. This substance, which Wright calls "opsonin," renders the bacteria in question less resistent. According to Wright, these opsonins, which are analogous to the other bacteriotropic substances (agglutinins, lysin and bactericidal substances) chemically unite with the bacteria in a manner entirely in harmony with Ehrlich's theory.

It appears that Neufeld and Rimpau, independently of Wright, showed that specific serum does not act upon the leucocytes in the sense that they are stimulated to take up more bacteria, but that the specific serum directly alters the bacteria and thus renders them more susceptible to phagocytosis. The property of the serum which so alters the bacteria through contact of opsonins that they are rendered susceptible to phagocytosis is called by Wright the opsonic power or opsonic index. The degree of opsonic index, therefore, in in direct relation to the number of bacteria taken up by the phagocytes of the blood. Wright found that under certain conditions, which may occur independently in the organism or be produced artificially by certain procedures, this opsonic power of human serum can be increased. The method for estimating the opsonic power or opsonic index is, according to Wright, as follows:

1. Take one volume of the blood of the individual and free it of formed elements.

2. Take from any healthy person an equal volume of formed blood elements which have been freed from blood serum by repeated washing with an isotonic sodium citrate solution and sodium chlorid solution.

3. An equal volume of a sodium chlorid suspension of the bacteria culture toward which the opsonic index is to be determined. The degree of concentration of the sodium chlorid solution used for the suspension of the bacteria and the age of the culture depend upon the species of bacteria.

The three volumes are mixed, then placed in the incubator at 37 degrees C. (98.6 degrees F.) for from 15 to 20 minutes. From this mixture smear preparations are made and stained with Leishmann's stain. The preparations are then placed under the microscope and the polynuclear phagocytes looked for. From 60 to 100 phagocytes are examined for the number of bacteria they have incorporated. The number of bacteria taken up by the phagocytes divided by the number of phagocytes counted Wright calls the phagocytic index.

To determine the opsonic power of a patient, the phagocytic index of a healthy individual, i. e. of a person who is free of the disease in question, is taken for comparison. The phagocytic index of the patient divided by that of the healthy person used for control gives the opsonic index. The degree of opsonic index, therefore, is in direct example, the phagocytic index of the patient's serum is 15 and that of the healthy person 10, then the opsonic index or opsonic power of the patient is 15÷10=1.5.

How may the opsonic index of an individual be influenced? If a definite amount of sodium chloride suspension of the bacteria in question (vaccine),—the amount varies according to the species of bacteria,—which has been heated for one hour at 60 degrees C. and thus killed is subcutaneously injected into an individual, examination of the opsonic power will show the following: In the first 24 hours or the first days the opsonic power of the individual falls to a lower point than it previously was. As a rule, the opsonic index remains for a shorter or longer time below normal, then it rises and for a certain time remains above normal whereby the size of the dose of vaccine injected is of importance. Then the index again sinks to a point which, however, is higher than before inoculation, and at this point the opsonic index remains for a certain time.

According to analogy with the results of Ehrlich's investigations upon inoculation with bacteriotoxins, Wright calls this state in which the opsonin content of the serum is low, i. e., when it has fallen, a negative phase, when it is high, i. e., increased, a positive phase.

Wright's experiences showed that in case the dose of the inoculated vaccine was small the negative phase was only slightly perceptible and lasted only a brief time, while the positive phase reached a higher degree and remained at this point for a longer time. If the injections of the vaccine were repeated during the negative phase, a cumulative action of the negative phase is obtained, so that the opsonic content of the serum is usually lower and at the same time the negative phase lasts longer. The reverse occurs when the inoculations are made during the positive phase, i. e., the opsonic index rises and usually remains high for a longer time.

In Wright's method of examining the opsonin content of the serum of the patient we have the possibility of controlling the state of the serum and of repeating the inoculations in the positive phase. According to Wright the clinical phenomena are always in harmony with the positive and negative phases. In the positive phase the pathologic manifestations diminish and disappear and the general condition is markedly improved. In the negative phase it is the reverse: immediately after each injection the clinical manifestations become worse; with each new inoculation this aggravation becomes less intense, less perceptible and lasts a shorter time and furthermore, its occurrence is postponed with each new inoculation.

In the practical employment of the opsonin theory, only those bacteria which produce the disease in the person in question are used. Let us assume that we have had before us a patient with acne, caused by staphyloccoci, who is to be treated by Wright's method. The pus from a ripe acne pustule is spread upon agar and from this a pure culture of staphylococcus obtained. A little blood is taken from the finger-tip of the patient by means of a sterile glass popette and centrifugated and the serum thus freed from formed elements. The opsonic index of the patient toward the staphylococci found in the acne pustule is then determined by the above method as soon as a pure culture is secured. As a rule the culture is ready after twenty-four hours and can then be used as vaccine. This is done in the following manner: from five to eight cubic centimeters of sterile physiologic sodium chlorid solution are poured into the test-tube containing the agar culture of staphylococcus; the cultrue growth on the agar is then rubbed off with a sterile glass rod, and mixed with the fluid. The saline suspension of cocci thus made is transferred to a second sterile test-tube and the open end of it drawn out into a capillary tube and sealed in the flame.

The strength of the vaccine used is determined by the number of cocci present in the vaccine. For this purpose one volume of the fresh vaccine is mixed with an equal volume of blood drawn from the finger-tip of some individual. From this mixture spread preparations are made and stained with Leishmann's solution. The red blood corpuscles and cocci in from ten to twenty microscope fields are counted. From the numbers found the ratio of cocci to red blood corpuscles in the mixture is determined. As 5,000,000 red blood corpuscles are present normally in one cmm. it is easy to determine how many cocci are present in one ccm. of the vaccine in question. The vaccine is now sterilized at 60 degrees C. for one hour in order to kill the cocci. Of course, a test for the presence of still living cocci should always be made before use of the vaccine. The total quantity of vaccine in the test tube is estimated by the following furmula: (in which *r* indicates half of the diameter of the vaccine fluid in the test-tube and *h* the height of the column of fluid) and 2.5 cmm. of pure lysol added to each cubic centimeter of the vaccine. The vaccine is now ready for use. The number of cocci in one cubic centimeter can be reckoned and correspondingly the dosage.

The doses of vaccine, which are injected once a week, vary according to the opsonic index. The doses of vaccine depend also upon the disease, i. e., the species of bacteria, so that in gonococcic or streptococcic affections entirely different doses must be employed.

In conclusion the author makes some criticisms: in counting the cocci taken up by the leucocytes for purpose of estimating the opsonic index and likewise in counting the bacteria in the vaccine made from the culture, there is the possibility of great subjective variations. In the method of estimating the opsonic index, however, it is not a question of absolute but of relative numbers. As the same investigator in counting the bacteria as well as the red blood corpuscles is liable to the same optical and subjective sources of error, the errors will not affect the final results. Therefore the preparations made for determining the opsonic index should always be examined by the same person.—The Post-Graduate, January, 1907.

